# Iron status of exclusively breastfed low-birth-weight infants born to anemic mothers and effect of maternal iron supplementation for 3 versus 6 months: A randomized double-blind placebo control trial

**DOI:** 10.3389/fped.2022.880431

**Published:** 2022-08-11

**Authors:** Tarannum Fatima, Mohammad Moonis Akbar Faridi, Geetika Srivastava

**Affiliations:** ERA’s Lucknow Medical College, Lucknow, Uttar Pradesh, India

**Keywords:** breastfeeding, low birth weight infant, anemia, lactation, iron supplementation

## Abstract

**Background:**

The effect of maternal iron supplementation during lactation on the iron status of exclusively breastfed low-birth-weight (LBW) infants is not known.

**Objective:**

(1) To find out the number of LBW exclusively breastfed infants having hemoglobin < 10.5 g/dL at 6 months when mothers received iron. (2) To find out the proportion of anemic infants when mothers received iron for 3 and 6 months.

**Design:**

The Clinical Trials Registry, India (CTRI) registered trial (CTRI/2018/08/015516) double-blind randomized control trial participants: A total of 80 anemic mothers and exclusively breastfed LBW infants.

**Intervention:**

A total of 80 anemic mothers and exclusively breastfed infants, birth weight 1,500–2,499 g, randomized into two groups of 40 each. Mothers received daily iron for 3 months and placebo for the next 3 months in group A and iron for 6 months in group B. Iron profile of mothers and infants measured at recruitment and 6 months.

**Results:**

In total, 26.6% infants developed anemia till 6 months of age, and number of anemic infants were similar whether mothers received iron for 3 (*n* = 9) or 6 months (*n* = 11). Hemoglobin (12.89 + 0.46 vs. 12.44 + 0.48 g/dL; *p* < 0.001) and serum ferritin (27.45 + 7.60 vs. 18.94 + 5.36 ng/mL; *p* < 0.001) were significantly higher in infants at 6 months of age whose mothers received iron for 6 months in comparison to 3 months. Conclusion: totally, 26.6% exclusively breastfed infants developed anemia till 6 months of age when mothers took iron; number of anemic infants were not different if mothers received iron for 3 or 6 months. A significant increase was noted in serum ferritin with slightly higher hemoglobin of infants when mothers received iron for longer duration.

**Clinical trial registration:**

[http://ctri.nic.in/Clinicaltrials/pubview.php], identifier [CTRI/2018/08/015516].

## Introduction

Iron deficiency is the most prevalent and treatable cause of anemia. The World Health Organization (WHO) published a Global Health Observatory Data in 2016 showing the population at risk of iron deficiency; women in reproductive age group (39%), pregnant women (46%), and children below 5 years of age particularly infants (42%) (1). It is worrying to see that the prevalence of anemia has spiked in India among children aged 6–59 months in the recent National Family Health Survey-5 (NFHS) conducted in the year 2019–2021 compared with NFHS-4 (2015–2016). There is 8.5% rise in the prevalence of anemia from 58.6% (NFHS-4) to 67.1% (NFHS-5), and rural children are doing poorer (68.3%) than their urban peers 64.2%). During the same period, a rise of 1.8% in the prevalence of anemia is seen in the pregnant women from 50.4 to 52.2%, but the difference in the rural and urban pregnant women is alarming (54.3 vs. 45.7%) (2). WHO recommends exclusive breastfeeding to infants for the first 6 months after birth for optimal growth and development (3). Having good bioavailability of the breast milk iron content varies widely during lactation (4). Term infants on exclusive breastfeeding do not require iron supplementation (5). Low birth weight (LBW) and preterm infants are born with lower iron stores compared with term infants (6). Exclusive breastfeeding, coupled with low iron stores, is not sufficient to meet the growing iron requirements and can cause iron deficiency in these infants. Iron supplementation is, therefore, recommended to LBW and preterm infants from 4 to 6 weeks of age (7). Iron supplementation, however, puts the infant at risk of gastrointestinal side effects, such as diarrhea, constipation, and loose stools, and poor compliance has also been reported (8). Recent evidence shows that oral iron administration induces pathogenic overgrowth and increases the virulence of prevalent enteric pathogens (9). Thus, an alternative approach can be adopted, wherein by replenishing maternal iron stores during lactation, the infant may acquire adequate amount of iron *via* breastfeeding catering to its iron requirements, as well as of its mother’s, bypassing the side effects of oral iron supplementation. Oral iron administration does cause gastrointestinal side effects in the lactating mothers (10), but some iron compounds have better tolerability and efficacy (11) and can be given to lactating mothers. This study has, therefore, been undertaken to assess the iron status of exclusively breastfed LBW infants born to anemic mothers who are supplemented with oral iron during lactation for 3 vs. 6 months with the following objectives.

### Objectives

(1) To find out the number of LBW exclusively breastfed infants developing anemia [hemoglobin (Hb) < 10.5 g/dL] by 6 months of age when their mothers receive oral iron supplementation.

(2) To find out proportion of anemic infants when mothers receive iron for 3 and 6 months, respectively.

(3) *Secondary objective:* To find out serum ferritin concentration in LBW infants at 6 months when mothers have received iron for 3 and 6 months, respectively.

## Materials and methods

The Clinical Trials Registry, India (CTRI)-registered double-blind randomized trial was carried in the Department of Pediatrics and Neonatology, in a teaching hospital from September 2018 to March 2020. Approval from the Institutional Ethics Committee for Human Research (ELMC/R_Cell/EC/2018/49) was obtained. Trial design was based on P (population) I (intervention) C (comparator) O (outcome) format.

### Participants

Informed consent was taken, and 80 anemic mothers (Hb 7–11.9 g/dL) and their singleton LBW infants weighing 1,500–2,499 g irrespective of the gestation and sex were recruited within 7 days of birth. Exclusive breastfeeding was started within the first hour of birth and continued till 6 months of age. Predominantly breastfed infants were also included in the study.

Exclusion criteria were as follows: mothers with the history of antepartum hemorrhage, eclampsia, or blood transfusion. Infants born with major congenital malformation, chromosomal anomalies, genetic and metabolic disorder; infants requiring blood transfusion, exchange transfusion, treated for sepsis, or initiated on formula feeding in early neonatal period.

### Randomization and blinding

Randomization of the subjects was performed by a computer-generated randomization table into two groups where mothers received either iron for 6 months or iron for 3 months followed by placebo for the next 3 months. The drug and placebo were coded in the randomization sequence as A or B and serially numbered in a randomization sequence from 1 to 80 by a third person not involved in the study, and key was kept by the same person. The allocation of mother–infant pair to intervention or control group was carried out by the serially numbered opaque sealed envelope (SNOSE) concealment technique. Randomization key was decoded in two steps. First, the serial numbers were converted to either A or B, and data were analyzed. After the statistical results were available, the second decoding was done; “A” included anemic mothers receiving iron for 3 months and placebo for the next 3 months (control group) and mothers in group “B” received iron for 6 months (intervention or study group). Thus, subjects, investigators, and data analyzers were not aware of the characteristics of the two groups until the results were revealed.

#### Iron capsules and placebo

Zuventus Healthcare, India provided samples of Ferrous ascorbate tablets (100 mg of elemental iron per tab). Iron Capsules and placebo(inert sugar) were prepared by pharmacy department. Both the drugs had same shape, color, and size. All mothers were given one iron capsule for daily consumption in the first 3 months followed by same iron dose to the intervention group or placebo to the control group for the next 3 months.

### Follow-up

Relevant history, height, weight, and BMI of mothers were recorded. Anthropometric parameters of the infant (i.e., weight, length, and head circumference) were recorded as per standard methods. Each mother and baby were followed up at 6 (±1), 10 (+1), 14 (±1), and 18 (±1) weeks and at 6 months (±1) of age. The mothers were counseled to practice exclusive breastfeeding and advised to take the prescribed drug daily. Empty capsule bottles were collected at each visit to check for compliance. Mothers who did not take the capsule consecutively for 2 weeks or >15% were considered “non-compliant.” All mothers were inquired for any adverse effects (i.e., constipation, loose stools, gastritis, vomiting, and black stools) at each visit. Infants were immunized as per the State Immunization Schedule, and their anthropometric parameters were recorded. The LBW infants should have received iron as per standard norms from the age of 6 weeks. To address this ethical issue, Hb of all infants was estimated at each follow-up. About 0.02 mL capillary blood was taken by heal prick, and Hb was estimated using the cyanmethemoglobin method. If the infant developed anemia (Hb < 10.5 g/dL), then iron supplementation was given at 2 mg/kg/day elemental iron for 2 months or till Hb reached ≥10.5 g/dL.

### Investigations

On recruitment, maternal and infant’s venous blood samples were collected. Maternal and infant’s Hb was estimated by automated hematology cell counter XS-800i (Sysmex Hemat Analyzer, Norderstedt, Germany). Serum was separated and stored at −20°C for estimation of serum ferritin and serum iron. At 6 months of age, venous blood was drawn from the mother–infant dyad to measure serum ferritin and serum iron. Ferritin was measured using ELISA kit–Calbiotech Ferritin EIA [The Calbiotech Inc., El Cajon, CA, United States, lot no. FR248T]. Quantitative C-reactive protein (CRP) was determined alongside serum ferritin to rule out false rise in the serum ferritin levels due to bacterial infection.

### Sample size

No study was available, which inquired effect of the iron supplementation to the anemic mothers on the hematological profile of their LBW infants. In a study by Hegde et al. (12), 26.8% late preterm infants were found anemic at 6 months of age irrespective of the iron supplementation to them at 2 or 4 mg/kg/day. We assumed this study closer to our objectives as iron supplementation to the lactating mothers may be a surrogate for iron supplementation to the LBW infants. Assuming the prevalence of anemia in the exclusively breastfed LBW infants to be approximately 30% when their mothers were supplemented with iron during lactation, and then 33 infants in each group (80% power; 5% significance level) would be sufficient by the following formula. Assuming a dropout rate of 15%, a total of 80 infants and their mothers were recruited with 40 infant–mother pairs in each group.


$$n=\frac{{\left(z_{\alpha }+z_{\beta }\right)}^{2}}{{\left[\ln\left(1-e\right)\right]}^{2}}\,\left[\frac{1-p_{1}}{p_{1}}+\frac{1-p_{2}}{p_{2}}\right]$$

*p*_1_ = 0.268 (26.8%); anemic children after iron supplementation.

*p*_2_ = 0.295 (29.5%); anemic children when mothers received iron.

*e* = 0.5 (*p*_2_/*p*_1_), clinically significant risk difference Type I error, α = 5%.

Type II error β = 20% for setting power of study 80% Data loss factor = 15%.

The sample size is calculated to be *n* = 40.

### Statistical analysis

Data were analyzed using the SPSS software (version 21.0). Mean, median, and interquartile range (IQR) were determined for quantitative variables, and qualitative variables were presented in numbers and percentage. Mean and standard deviation (SD) values of serum iron, serum ferritin at recruitment and 6 months were compared using the paired *t*-test. The chi-square was applied to compare the proportion of qualitative variables between the groups. One-way ANOVA was applied to test for serum iron parameters for development of anemia over time. A correlation between mothers’ serum ferritin with that of infants was measured by the Pearson correlation coefficient. *P*-value < 0.05 was considered significant.

### Definitions

#### Anemia

Hb of <12 g/dL in lactating women and <10.5 g/dL during infancy (13).

#### Iron deficiency state

Serum ferritin level of <12 ng/mL (13).

#### Low birth weight

Absolute birth weight of <2,500 g irrespective of the gestational age. Very low birth weight (VLBW) is birth weight <1,500 g (14).

#### Preterm

Babies born alive before completed 37 weeks of pregnancy (15).

#### Small for gestational age

Fetus or newborn with birth weight below 10th percentile for the infant’s gestational age and sex (16).

#### Appropriate for gestational age

Fetus or newborn with the birth weight between 10th and 90th percentiles for the infant’s gestational age and sex (16).

#### Exclusive breastfeeding

Exclusive breastfeeding is defined as no other food or drink, not even water, except breast milk (including expressed breast milk), but allows the infant to receive oral rehydration solution (ORS), drops, and syrups (vitamins, minerals, and medicines) (17).

#### Predominantly breastfed

“Predominant breastfeeding” means that the infant’s predominant source of nourishment has been breast milk (including milk expressed or/from a wet nurse) as the predominant source of nourishment). However, the infant may have received liquids (i.e., water and water-based drinks and fruit juice), ritual fluids and ORS, drops, or syrups (i.e., vitamins, minerals, and medicines) (17).

## Results

**FIGURE 1:**
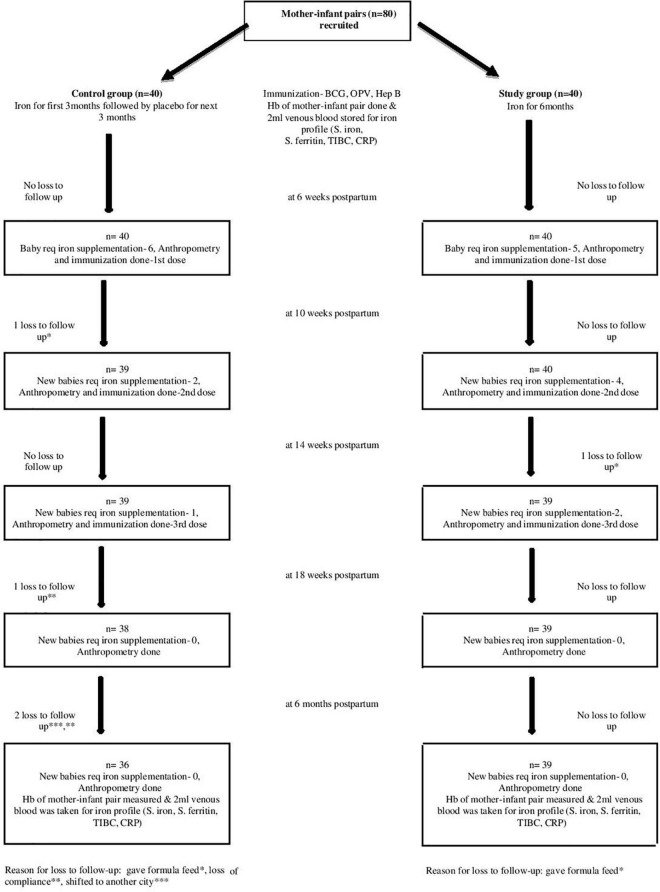
Study flow chart.

Eighty anemic mothers and their LBW infants were randomized into two groups in a block of 1:1. Mothers received oral iron daily for 3 months and placebo for the next 3 months in the control group or iron alone for 6 months in the intervention group during lactation. Total 75 mother–infant pairs (36 in control and 39 in intervention groups), completed follow-up till 6 months of age (Figure 1). The baseline characteristics of the mothers (i.e., age, BMI, gravidity, parity, and mode of delivery) and their infants (i.e., birth weight, sex, gestational age, and anthropometrical parameters) were comparable in both the groups (Table 1). Mean Hb (10.08 ± 0.65 vs. 10.02 ± 0.63 g/dL; *p* = 0.678) and serum ferritin (9.87 ± 0.90 vs. 9.65 ± 1.23 ng/mL; *p* = 0.394) of the mothers in the control and intervention groups, respectively, were not different and were consistent with the iron deficiency state. Similarly Hb (18.14 ± 1.3 g/dL) and serum ferritin (128.64 ± 40.28 ng/mL) of the LBW infants in the control group were similar to Hb (18.15 ± 1.23 g/dL) and serum ferritin (128.74 ± 44.75 ng/mL) of the infants in the intervention group on recruitment (Table 2). Further after excluding subjects due to loss to follow-up (*n* = 5), no significant difference was observed in the baseline profile of the remaining 75 mother–infant dyads in the two groups.

**TABLE 1:** Baseline characteristics of mothers and infants at recruitment.

Maternal characteristics		Control group		Study group		*P-value*
		No.	*n* = 36%	No.	*n* = 39%	
Age (years)	20–25	17	44.44%	21	53.84%	0.301
	26–30	18	52.79%	15	38.46%	
	31 and above	1	2.77%	3	7.70%	
Weight (kg)	50–55	25	69.44%	26	66.66%	0.328
	56–60	10	27.77%	9	23.07%	
	>60	1	2.79%	4	10.27%	
Height (cm)	150–155	20	55.55%	22	56.41%	0.59
	156–160	14	38.88%	16	41.02%	
	>160	2	5.57%	1	2.57%	
BMI (kg/m^2^)	<20	1	2.77%	0	0.00%	0.602
	20–24.9	32	88.88%	36	92.30%	
	>25	3	8.35%	3	7.70%	
Mode of delivery	LSCS	10	27.77%	16	41.02%	0.237
	VD	26	72.23%	23	58.98%	
Parity	PRIMI	18	50.00%	18	46.15%	0.442
	PARA 2	16	44.44%	17	43.58%	
	PARA 3	2	5.56%	4	10.27%	
**Infant characteristics**						
Sex	Male	17	47.22%	14	35.89%	0.256
	Female	19	52.78%	25	64.11%	
Birth weight (gm)	1,500–1,999	1	2.77%	3	7.69%	0.305
	2,000–2,499	35	97.23%	36	92.31%	
Period of gestation	PRETERM (<37 weeks)	16	44.44%	14	35.89%	0.491
	TERM (<37 weeks)	20	55.56%	25	64.11%	
Preterm	AGA	15	93.75%	14	100.00%	0.356
	SGA	1	6.25%	0	0.00%	
Term	AGA	13	65.00%	16	64.00%	0.99
	SGA	7	35.00%	9	36.00%	
Weight/age	<10th Perc	8	22.22%	7	17.94%	0.785
	10th–90th Perc	28	77.78%	32	82.06%	
Length/age	10th–90th Perc	36	100.00%	39	100.00%	NA
Head circumference/age	10th–90th Perc	36	100.00%	39	100.00%	NA

LSCS, lower segment C-section; VD, vaginal delivery; AGA, appropriate for gestational age; SGA, small for gestational age; BMI, body mass index; Perc, percentile.

**TABLE 2:** Baseline hematological parameters of mothers and infants at recruitment.

Subjects	Control group (*n* = 36)		Study group (*n* = 39)		*P-value*
	Mean	SD	Mean	SD	
**Maternal parameters**					
Hb (g/dL)	10.08	0.65	10.02	0.63	0.678
PCV (%)	33.55	2.49	33.61	2.51	0.908
MCH (pg)	22.63	1.54	22.46	1.54	0.628
S. FERRITIN (ng/mL)	9.87	0.9	9.65	1.23	0.394
CRP (pg/mL)	<5	0	<5	0	NA
**Infant’s parameters**					
Hb (g/dL)	18.14	1.3	18.15	1.23	0.958
PCV (%)	51.65	3.25	51.04	3.62	0.432
MCH (pg)	34.12	2.19	34.54	1.8	0.351
S. FERRITIN (ng/mL)	128.64	40.28	128.74	44.75	0.992
CRP (pg/mL)	<5	0	<5	0	NA

Hb, hemoglobin; PCV, packed cell volume; MCH, mean corpuscular hemoglobin; CRP, C- reactive protein.

Notably, 20 out of 75 (26.7%) exclusively breastfed LBW infants developed anemia (Hb < 10.5 g/dL) till 6 months of age in two groups. In the control group, six infants at 6 weeks, two infants at 10 weeks, and one infant at 14 weeks (total 9/36, 25% infants) developed anemia on follow-up. In the intervention group, the number of anemic infants were 5, 4, and 2 (total 11/39, 28.2% infants) during the same period and were comparable in the two groups. None of the infants was found anemic at 18 weeks and at 6 months of age. Thus, a total of 26.7% (*n* = 20/75) infants in the two groups developed anemia and required iron supplementation at 6, 10, and 14 weeks on follow-up till 6 months of age (Figure 1).

**FIGURE 2:**
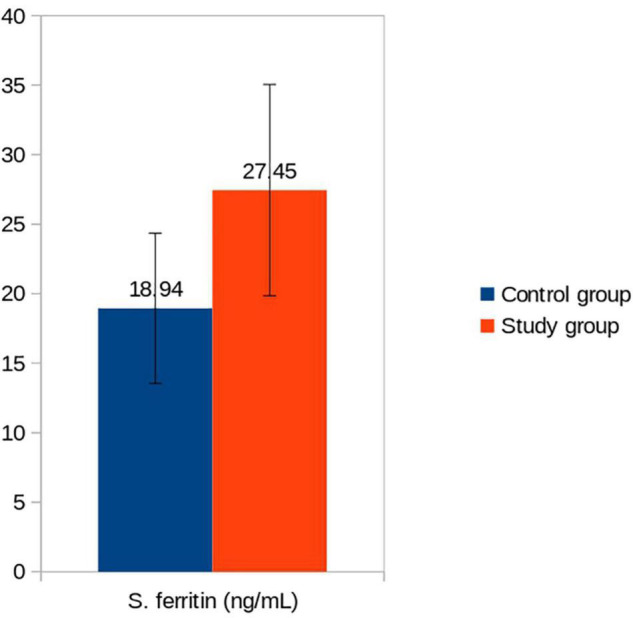
Serum ferritin (ng/mL) of infants in control vs. study groups at 6 months. Serum ferritin: Control vs. Study group (*p* < 0.001).

Exclusively breastfed infants of the mothers receiving daily iron for 6 months had higher serum ferritin concentration (27.45 ± 7.60 vs. 18.94 ± 5.36 ng/mL; *p* < 0.001) and Hb (12.89 ± 0.46 vs. 12.44 ± 0.48 g/dL; *p* < 0.001) levels at 6 months of age compared with the infants whose mothers were supplemented with daily iron for 3 months only (Table 3). Only one (1.3%) out of 75 infants had iron deficiency state (serum ferritin < 12 ng/mL) at 6 months of age. This infant belonged to control group. All infants in the study group had serum ferritin ≥15 ng/mL (Table 4). Iron supplementation to anemic mothers during lactation raised serum ferritin and Hb in the exclusively breastfed LBW infants more so when it was done for 6 months compared with 3 months (Figure 2 and Table 5). The anthropometry parameters were similar in two groups at 6 months of age.

**TABLE 3:** Hematological parameters of mothers and infants at 6 months.

Subjects	Control group (*n* = 36)		Study group (*n* = 39)		*P-value*
	Mean	SD	Mean	SD	
**Maternal parameters**					
Hb (g/dL)	12.76	0.31	13.17	0.27	<0.001
PCV (%)	38.86	1.36	40.13	1.12	<0.001
MCH (pg)	29.53	0.6	30.83	0.94	<0.001
Serum ferritin (ng/mL)	84.77	6.87	115.22	6.95	<0.001
CRP (pg/mL)	<5	0	<5	0	NA
**Infants’ parameters**					
Hb (g/dL)*	12.44	0.48	12.89	0.43	<0.001
PCV (%)	36.79	1.9	38.57	1.28	<0.001
MCH (pg)	25.62	1.06	27.01	0.58	<0.001
Serum ferritin (ng/mL)*	18.94	5.36	27.45	7.6	<0.001
CRP (pg/mL)	<5	0	<5	0	NA

Hb, hemoglobin; PCV, packed cell volume; MCH, mean corpuscular hemoglobin; CRP, C-reactive protein. The parameters symbolized by an asterisk are the outcomes of this study. The *p*-value is significant at <0.005.

**TABLE 4:** Comparison of serum ferritin concentration in babies of both groups at 6 months.

Serum ferritin (ng/mL)	Control group (*n* = 36)		Study group (*n* = 39)		χ^2^	*P-value*
	No.	%	No.	%		
<12	1	2.80%	0	0.00%	1.1	0.295
>12	35	97.20%	39	100.00%		
<15	6	16.70%	0	0.00%	7.07	0.008
>15	30	83.30%	39	100.00%		

The *p*-value is significant at <0.005.

**TABLE 5:** Hemoglobin (g/dL) of infants at 6 months and number of infants developing anemia till 6 months.

Subjects	Control group (*n* = 36)		Study group (*n* = 39)		*P-value*
	Mean	SD	Mean	SD	
Hb (g/dL)	12.44	0.48	12.89	0.43	<0.001
**No. of anemic infants till 6 months**					
*n* =	9		11		NA

The *p*-value is significant at <0.005.

The gestation and birth weight and hematological parameters of the infants on recruitment, who developed anemia during follow-up and required iron supplementation, were also comparable in the two groups but their mean serum ferritin levels (control group 74.20 ± 11.92 ng/mL and study group 85.0 ± 30.99 ng/mL) (Table 6) were lower than the mean ferritin concentration in each group (128.64 ± 40.28 ng/mL in control group; 128.74 ± 44.75 ng/mL in study group) (Table 2). Lower iron endowments at birth might have predisposed these infants to develop anemia during follow-up.

**TABLE 6:** Baseline hematological parameters of infants at recruitment who developed anemia during follow up and received iron supplementation.

Hematological parameters	Control group (*n* = 9)		Study group (*n* = 11)		*t*-value	*P-value*
	Mean	SD	Mean	SD		
Hb (g/dL)	16.84	0.52	17.19	0.81	−1.09	0.292
Serum Ferritin (ng/mL)	74.20	11.92	85.00	30.99	−0.74	0.470
CRP (pg/mL)	<5	0	<5	0	NA	NA
**Anthropometrical parameters**						
Period of gestation						
32–34 weeks	3	NA	2	NA	NA	NA
35–36 weeks	6		8			
>37 weeks	0		1			
Birth weight						
1,500–1,999 gm	1	NA	3	NA	NA	NA
2,000 –2,499 gm	8		8			

Hb, hemoglobin; CRP, C-reactive protein.

The maternal iron status too markedly improved irrespective of the duration of iron supplementation, and the serum ferritin concentration rose from the deficient values of 9.87 ± 0.9 and 9.65 ± 1.23 ng/mL at recruitment (Table 2) to 84.77 ± 6.87 and 115 ± 6.95 ng/mL at 6 months in the control and study groups, respectively (Table 3). Further, the rise in the serum ferritin concentration was significantly more (*p* < 0.001) in the mothers who received iron supplementation for 6 months. The maternal serum ferritin levels at delivery and at 6 months into lactation had overall positive correlation with that of the infants at birth and at 6 months of age. However, maternal serum ferritin concentration at recruitment had over all negative correlation with that of baby’s at 6 months and difference between two groups was significant. This was because of the fact that infants used up their iron endowment and maternal iron supplementation for 6 months in the study group helped these infants to maintain better ferritin concentration (Table 7).

**TABLE 7:** Correlation of maternal serum ferritin concentration with those of infants at recruitment and 6 months.

Ferritin concentration (ng/mL)	Overall		Control group		Study group	
	Pearson correlation	*P-value*	Pearson correlation	*p*-value	Pearson correlation	*P-value*
Maternal and baby’s ferritin at recruitment	0.490	<0.001	0.465	0.004	0.513	0.001
Maternal and baby’s ferritin at 6 months	0.429	<0.001	−0.312	0.064	−0.133	0.421
Maternal ferritin at recruitment to baby’s ferritin at 6 months	−0.335	0.003	−0.243	0.154	−0.381	0.017

The *p*-value is significant at <0.005.

Oral iron supplementation to the mothers caused gastritis and constipation, but none of them had diarrhea or vomiting. Notably, 20 and 15% mothers complained of heart burning and retrosternal discomfort at first follow-up 6 weeks after initiation of oral iron intake in the control and study groups, respectively. Gastritis was found in 10.3 and 10% mothers in the respective groups on second follow-up at 10 weeks. After 14 weeks, 2.6% mothers in the control group and 10.3% mothers in the study group had symptoms of gastritis. The gastritis was present in 2.6 and 5.1% mothers in the respective groups at 18 weeks. Although number of the mothers complaining gastritis was more in the study group at 14 and 18 weeks, while they were receiving iron during this period unlike control group where a placebo was given, the difference was not significant (at 14 weeks *p* = 0.240, at 18 weeks *p* = 0.513). Constipation was seen in one mother of the control group only at each follow-up; it was revealed that she suffered from chronic constipation. Constipation was not found in any mother belonging to the study group. No mother stopped iron intake on account of intolerability.

## Discussion

This study comprised 80 anemic mothers who were randomized to receive oral iron supplementation daily for either 3 (control group) or 6 months (study group) during lactation and their exclusively breastfed LBW infants. Out of 80 mother–infant pairs, 75 of them (36 in control and 39 in study groups) completed the study. Required sample size was achieved. About 26.7% infants developed anemia till 6 months of age, and the number of infants was not different in two groups. Hb and serum ferritin were significantly higher in the infants at 6 months of age whose mothers received iron for 6 months compared with 3 months. Furthermore, none of the infants developed iron-deficient (ID) state when mothers took iron for 6 months. The maintenance of iron status and prevention of anemia in almost three-fourth of infants at 6 months of age without iron supplementation must be due to higher iron content of the breast milk and transfer of iron to them *via* breastfeeding only. The CRP concentration was within normal limits.

Iron is the most abundant trace element in the human body and plays a critical role in neuro-cognitive development of the infants and young children. It is also a cofactor in various enzymatic reactions and neurotransmitter synthesis such as tryptophane hydroxylase (serotonin) and tyrosine hydroxylase (norepinephrine); both help in mood elevation (18). Iron remains in the human body bound to two major iron-binding proteins, transferrin and ferritin. In the breast milk, iron is reversibly bound to a specific iron-binding protein, lactoferrin. Inside the body, 60% iron is bound to Hb and 20% iron is stored as ferritin. Less than 1% iron circulates in the blood bound to transferrin (19). Ferritin levels are an important marker for iron status (19, 20). A healthy fetus acquires 1.35 mg/kg iron in the last trimester of pregnancy maintaining an average iron content of 75 mg/kg body weight (21). Breast milk iron contents coupled with iron stores of the infant, acquired near term gestation during intrauterine life, and accumulated in the neonatal period by red cell destruction are sufficient to maintain normal iron metabolism in the first 6 months of life in exclusively breastfed term AGA babies (5). Maternal iron deficiency (ID) decreases body iron contents of the infant at birth in linear proportion to serum ferritin (22). Similarly, preterm infants are born with reduced iron stores as iron transfer to the fetus takes place near term. Severe ID in the mother, in contrast, adversely affects breast milk iron contents also (23, 24). However, ID is not evident in these infants at birth; their Hb and serum ferritin remain within normal limits. These infants use up their iron stores by 2–3 months of age as a result of comparatively rapid body growth and expansion of the vascular compartment. Low iron contents in the breast milk, which vary during lactation and are affected by severity of maternal ID, does not rescue the situation either (4, 6). Iron deficiency and iron deficiency anemia ensues. Mehta et al. reported that 76.9% LBW infants develop anemia between 10 and 6 months of age and ID (serum ferritin < 10 ng/mL) is seen in 32.2% infants at 6 months if they are not supplemented with iron (25). It is recommended that these infants should receive iron from the age of 6–8 weeks in order to replenish body iron stores before erythropoiesis starts so that ID and IDA are prevented. All LBW infants in our study had normal Hb and serum ferritin levels at birth though their mothers were iron deficient and anemic at the time of delivery. They were exclusively breastfed and not supplemented with iron; instead iron was given to their mothers. It was remarkable to see that about three-fourth LBW infants did not develop IDA, and ID was seen in only one infant at 6 months. The Hb and serum ferritin concentration of both the mothers and their infants were significantly higher in 6 months as compared with 3-month iron supplementation group. It could be possible only when sufficient amount of iron was secreted in the breast milk of iron-supplemented mothers, and their exclusively breastfed infants could ingest and absorb iron from the breast milk. Iron metabolism is dynamically controlled in the body, but studies in the human infants and experimental animals have shown that iron transporters such as divalent metal-ion transporter 1 (DMT 1) and ferroportin are dysregulated in the early infancy, and iron homeostasis is either absent or limited (26). Thus, LBW and preterm infants absorb iron present in the breast milk or formula milk independent of serum ferritin and soluble transferrin receptors in early months of life, and this helps them to build iron stores.

We assumed that there was an upregulation of transferrin receptors in the mammary glands of the anemic lactating mothers resulting in an increased uptake of transferrin bound iron from the maternal blood flowing through the mammary gland. Severe ID and IDA in mothers lead to increased expression of transferrin receptors in the breast tissues, which trap more iron and secrete iron in the breast milk (27).

Iron is stored inside the mammary epithelium as ferritin and in the breast milk as lactoferrin. Secretion of iron in the breast milk occurs *via* iron–lactoferrin complex instead of direct transmembrane transport as lactoferrin is the only known iron-binding protein found in the human milk (28). It has been reported that prevalence of anemia in the LBW infants at the age of 6 months was 26.8% when they were supplemented with oral iron either at 2 or 4 mg/kg/day (12). Iron supplementation to anemic mothers also achieved the same prevalence of anemia in the LBW infants. The oral iron was well tolerated by the mothers, and their Hb and iron status markedly improved irrespective of the duration of iron supplementation. This was a bonus.

Iron content of the breast milk ranges from 0.1 to 1.6 mg/L across different stages of lactation (4). Curran et al. (4) found that iron content of colostrum was higher (1.0 mg/L), whereas mature milk had slightly lower iron concentration of about 0.20–0.80 mg/L. Wide variation is observed in the breast milk iron contents among different ethnicities with higher values in Indian (1.11 mg/L) and Malayan women (1.16 mg/L) compared with the Chinese mothers (0.81 mg/L) (29). Period of gestation at delivery also affects iron contents in the breast milk. Mothers giving birth to preterm babies have higher breast milk iron concentration (39.06 ± 10.78 μmol/L) compared with their peers delivering at term (32.79 ± 14.17 μmol/L) (30). Iron in the breast milk has higher bioavailability of about 49% by radio isotope technique and 16–25% by stable isotope method, which is largely due to high lactoferrin content of the breast milk (31). It can be hypothesized that iron absorption by the infants through breast milk *via* maternal supplementation will be superior to direct iron supplementation to them because of better bioavailability without adverse effects. Lactoferrin is a multifunctional iron-binding glycoprotein that reversibly binds one ferric ion and remains 10% saturated in the human milk.

Multiple studies have been carried out to assess the impact of maternal nutrition and intake of micronutrient rich diet on the breast milk composition (32). Recently, Sanchez et al. (33) has reported that iron contents in the breast milk of Galician lactating women are higher if diet is rich in iron and selenium. However, few studies in the past have reported no relation between maternal dietary iron intake and iron contents in the breast milk (34). Baykan et al. (35) supplemented iron to non-anemic breastfeeding mothers and found that iron supplementation to the mothers during the first 4 months of lactation had no impact on the serum iron and ferritin levels of both the mothers and their exclusively breastfed term infants. However, maternal iron supplementation increases total iron ligands in breast milk, as measured by total iron-binding capacity and increased proportion of lactoferrin in the total protein secreted (36).

It is noted that oral iron supplementation to the infants increases the risk of bacterial infections and alteration in the gut microbiota presenting as constipation, diarrhea, and black stools (37). Study conducted in Hacettepe University reported that 44.4% infants receiving iron supplementation suffered from the GI side effects (38). Poor compliance (38%) has also been reported, putting the infants at risk of anemia (8). Maternal iron supplementation to anemic mothers during lactation seems to be an exciting strategy for safely preventing ID and IDA in the LBW infants and simultaneously treating ID in them.

Only 26.7% LBW infants developed anemia by 6 months of age. The ID manifests in three stages. Stage 1 is characterized by iron depletion and serum ferritin, and transferrin saturation is decreased. In stage 2, a stage of iron-deficient erythropoiesis, the soluble transferrin receptors in the plasma, and zinc protoporphyrin in the RBCs also increase. Stage 3 of ID heralds severe iron deficiency and is characterized by fall in Hb and changes in the red blood cells morphology such as microcytosis and hypochromia (39). Iron endowment of these infants at birth was lower than the group they belonged to and breast milk iron contents achieved after maternal iron supplementation could not prevent development of anemia.

The limitations of this study were as follows: (i) LBW infants constituted a diverse group having variability in their iron stores and iron metabolism, which were not accounted for separately, (ii) breast milk iron and lactoferrin levels were also not determined, and (iii) sample size was calculated on an assumption that maternal iron supplementation would be a surrogate to infant oral iron administration. The strength of the study is that it is a well-designed double-blinded randomized control trial registered with CTRI, and we were able to achieve the desired sample size on follow-up.

## Conclusion

Supplementation of anemic mothers with iron during lactation prevented anemia in 73.4% of exclusively breastfed LBW infants. The number of anemic infants was similar irrespective of the duration of iron supplementation for 3 or 6 months. Significantly higher serum ferritin concentration and Hb levels were observed in infants whose mothers received iron for longer duration. Oral supplementation of iron to anemic mothers during lactation also improved their own iron status and Hb more so if iron supplementation was carried out for 6 months.

## Data availability statement

The original contributions presented in this study are included in the article/supplementary material, further inquiries can be directed to the corresponding author.

## Ethics statement

The studies involving human participants were reviewed and approved by Institutional Ethics Committee for Human Research (Era Medical College and Hospital, Lucknow-ELMC/R_Cell/EC/2018/49). Written informed consent to participate in this study was provided by the participants or their legal guardian/next of kin.

## Author contributions

MF contributed to the conception and design of the study. TF and GS organized the database. TF performed the statistical analysis and wrote the first draft of the manuscript. MF, TF, and GS wrote sections of the manuscript, contributed to the manuscript revision, read, and approved the submitted version.

## References

[1] World Health Organization. *Global Health Observatory Data Repository: Anaemia in Children <5 Years by Region.* (2016). Available online at: http://apps.who.int/gho/data/view.main.ANEMIACHI LDRENv?lang=en (accessed on July 2, 2020).

[2] National Family Health Survey of India-5 on Incidence of Anemia. *Compendium of Factsheets.* (2019-2021). Available online at: http://main.mohfw.gov.in (accessed on May 26, 2022).

[3] World Health Organization, UNICEF. *Global Strategy for Infant and Young Child Feeding.* Geneva: World Health Organization (2019).

[4] Curran JS Barness LA. The feeding of infants and children. In: Behrman RE Kleigman RM Jenson HB editors. *Nelson Textbook of Pediatrics.* Philadelphia, PA: WB Saunders (2004). p. 149–69.

[5] Raj S Faridi MMA Rusia U Singh O. A prospective study of iron status in exclusively breastfed term infants up to 6 months of age. *Int Breastfeed J.* (2008) 3:3. 10.1186/1746-4358-3-3 18312681 PMC2277383

[6] Moreno-Fernandez J Ochoa JJ Latunde-Dada GO Diaz-Castro J. Iron deficiency and iron homeostasis in low birth weight preterm infants: a systematic review. *Nutrients.* (2019) 11:1090. 10.3390/nu11051090 31100900 PMC6566715

[7] Berglund S Domellof M. Meeting iron needs for infants and children. *Curr Opin Clin Nutr Metab Care.* (2014) 17:267–72. 24535217 10.1097/MCO.0000000000000043

[8] Powers JM Daniel CL McCavit TL Buchanan GR. Deficiencies in the management of iron deficiency anemia during childhood. *Pediatr Blood Cancer.* (2016) 63:743–5. 26728130 10.1002/pbc.25861 PMC4755821

[9] Kortman GA Boleij A Swinkels DW Tjalsma H. Iron Availability increases the pathogenic potential of *Salmonella typhimurium* and other enteric pathogens at the intestinal epithelial interface. *PLoS One.* (2012) 7:e29968. 10.1371/journal.pone.0029968 22272265 PMC3260200

[10] Tolkien Z Stecher L Mander AP Pereira DI Powell JJ. Ferrous sulfate supplementation causes significant gastrointestinal side-effects in adults: a systematic review and meta-analysis. *PLoS One.* (2015) 10:e0117383. 10.1371/journal.pone.0117383 25700159 PMC4336293

[11] Martinez Frances A Leal Martinez-Bujanda J. Efficacy and tolerability of oral iron protein succinylate: a systematic review of three decades of research. *Curr Med Res Opin.* (2020) 36:613–23. 10.1080/03007995.2020.1716702 31944128

[12] Hegde A. *Efficacy of Iron Prophylaxis (2 mg/kg/day vs. 4 mg/kg/day) for Preventing Iron Deficiency in Late Preterm Infants: A Randomized Controlled Trial.* Ph.D. Thesis. Delhi: University College of Medical Sciences and GTB Hospital (2019).

[13] WHO. Haemoglobin Concentrations for the Diagnosis of Anaemia and Assessment of Severity. Geneva: WHO (2017).

[14] WHO. *Newborns With Low Birth Weight.* (2017). Available online at: https://www.who.int/data/nutrition/nlis/info/low-birth-weight (accessed November 26, 2017).

[15] WHO. *Preterm Birth.* (2017). Available online at: http://www.who.int/mediacentre/factsheets/fs363/en/ (accessed on November 26, 2017).

[16] Onis M Habicht JP. Anthropometric reference data for international use: recommendations from a World Health Organization expert committee. *Am J Clin utr.* (1996) 64:650–8. 10.1093/ajcn/64.4.650 8839517

[17] WHO. *The World Health Organization’s Infant Feeding Recommendation.* (2017). Available online at: https://www.who.int/news-room/fact-sheets/detail/infant-and-young-child-feeding (accessed November 23, 2017).

[18] Rao R Tkac I Townsend EL Gruetter R Georgieff MK. Perinatal iron deficiency alters the neurochemical profile of the developing rat hippocampus. *J Nutr.* (2003) 133:3215–21. 14519813 10.1093/jn/133.10.3215

[19] McDermid JM Lonnerdal B. Iron. *Adv Nutr.* (2012) 3:532–3. 22797989 10.3945/an.112.002261 PMC3649722

[20] Knovich MA Storey JA Coffman LG Torti SV Torti FM. Ferritin for the clinician. *Blood Rev.* (2009) 23:95–104. 18835072 10.1016/j.blre.2008.08.001 PMC2717717

[21] Siddappa AM Rao R Long JD Widness JA Georgieff MK. The assessment of newborn iron stores at birth: a review of the literature and standards for ferritin concentrations. *Neonatology.* (2007) 92:73–82. 10.1159/000100805 17361090 PMC2863301

[22] Singla PN Tyagi M Shankar R Dash D Kumar A. Fetal iron status in maternal anemia. *Acta Paediatr.* (1996) 85:1327–30. 8955460 10.1111/j.1651-2227.1996.tb13919.x

[23] Kumar A Rai AK Basu S Dash D Singh JS. Cord blood and breast milk iron status in maternal anemia. *Pediatrics.* (2008) 121:e673–7. 18310187 10.1542/peds.2007-1986

[24] Widen EM Bentley ME Kayira D Chasela CS Daza EJ Kacheche ZK. BAN study team. changes in soluble transferrin receptor and hemoglobin concentrations in Malawian mothers are associated with those values in their exclusively breastfed, HIV-exposed infants. *J Nutr.* (2014) 144:367–74. 10.3945/jn.113.177915 24381222 PMC3927549

[25] Mehta M Faridi MMA Sharma S Singh O Sharma AKA. Prospective study of iron status of exclusively breastfed infants weighing 1800-2499g at birth and correlation with breast milk lactoferrin. *Int J Pediatr Child Health.* (2016) 4:42–51.

[26] Lonnerdal B. Development of iron homeostasis in infants and young children. *Am J Clin Nutr.* (2017) 106(Suppl. 6):1575S–80S. 10.3945/ajcn.117.155820 29070561 PMC5701726

[27] Fransson GB Agarwal KN Gebre-Medhin M Hambreus L. Increased breastmilk iron in severe maternal anemia: physiological ‘trapping’ or leakage? *Acta Paediatr Scand.* (1985) 74:290–1. 3993377 10.1111/j.1651-2227.1985.tb10967.x

[28] Sigman M Lonnerdal B. Response of rat mammary gland transferrin receptors to maternal dietary iron during pregnancy and lactation. *Am J Clin Nutr.* (1990) 52:446–50. 10.1093/ajcn/52.3.446 2393007

[29] Loh TT Sinnathuray TA. Haematological data and milk iron in Malaysian women. *Aust N Z J Obstet Gynaecol.* (1971) 11:254–8. 10.1111/j.1479-828x.1971.tb00488.x 5289727

[30] Ejezie F Nwagha U Ikekpeazu E Ozoemena O Onwusi E. Assessment of iron content of breast milk in preterm and term mothers in enugu urban. *Ann Med Health Sci Res.* (2011) 1:85–90. 23209959 PMC3507087

[31] Abrams SA. Using stable isotopes to assess mineral absorption and utilization by children. *Am J Clin Nutr.* (1999) 70:955–64. 10584039 10.1093/ajcn/70.6.955

[32] Bravi F Wiens F Decarli A Dal Pont A Agostoni C Ferraroni M. Impact of maternal nutrition on breast- milk composition: a systematic review. *Am J Clin Nutr.* (2016) 104:646–62. 27534637 10.3945/ajcn.115.120881

[33] Sanchez C Fente C Barreiro R Lopez-Racamonde O Cepeda A Regal P. Association between breast milk mineral content and maternal adherence to healthy dietary patterns in Spain: a transversal study. *Foods.* (2020) 9:659. 10.3390/foods9050659 32443751 PMC7278811

[34] Mello-Neto J Rondo PH Oshiiwa M Morgano MA Zacari CZ dos Santos ML. Iron supplementation in pregnancy and breastfeeding and iron, copper and zinc status of lactating women from a human milk bank. *J Trop Pediatr.* (2013) 59:140–4. 10.1093/tropej/fms055 23070740

[35] Baykan A Yalgin SS Yurdakok K. Does maternal iron supplementation during the lactation period affect iron status of exclusively breast-fed infants? *Turk J Pediatr.* (2006) 48:301–7. 17290563

[36] Keikha M Shayan-Moghadam R Bahreynian M Kelishadi R. Nutritional supplements and mother’s milk composition: a systematic review of interventional studies. *Int Breastfeed J.* (2021) 16:1. 33397426 10.1186/s13006-020-00354-0 PMC7780633

[37] Cancelo-Hidalgo MJ Castelo-Branco C Palacios S Haya-Palazuelos J Ciria-Recasens M Manasanch J Tolerability of different oral iron supplements: a systematic review. *Curr Med Res Opin.* (2013) 29:291–303. 23252877 10.1185/03007995.2012.761599

[38] Yurdakok K Temiz F Yalgin SS Gumruk F. The efficacy of daily and weekly iron supplementation on iron status in exclusive breast-fed infants. *J Pediatr Hematol Oncol.* (2004) 26:284–8. 10.1097/00043426-200405000-00005 15111779

[39] Domellof M Braegger C Campoy C Colomb V Decsi T Fewtrell M ESPGHAN committee on nutrition. Iron requirements of infants and toddlers. *J Pediatr Gastroenterol Nutr.* (2014) 58:119–29. 10.1097/MPG.0000000000000206 24135983

